# Protease expression in the human and rat cumulus–oocyte complex during the periovulatory period: a role in cumulus–oocyte complex migration^†^

**DOI:** 10.1093/biolre/ioae108

**Published:** 2024-07-17

**Authors:** Ketan Shrestha, Muraly Puttabyatappa, Michelle A Wynn, Patrick R Hannon, Linah F Al-Alem, Katherine L Rosewell, James Akin, Thomas E Curry

**Affiliations:** Department of Obstetrics and Gynecology, Chandler Medical Center, University of Kentucky, Lexington, KY, USA; Department of Obstetrics and Gynecology, Chandler Medical Center, University of Kentucky, Lexington, KY, USA; Department of Obstetrics and Gynecology, Chandler Medical Center, University of Kentucky, Lexington, KY, USA; Department of Obstetrics and Gynecology, Chandler Medical Center, University of Kentucky, Lexington, KY, USA; Department of Obstetrics and Gynecology, Chandler Medical Center, University of Kentucky, Lexington, KY, USA; Department of Obstetrics and Gynecology, Chandler Medical Center, University of Kentucky, Lexington, KY, USA; Bluegrass Fertility Center, Lexington, KY, USA; Department of Obstetrics and Gynecology, Chandler Medical Center, University of Kentucky, Lexington, KY, USA

**Keywords:** ovary, cumulus, granulosa cell, oocyte, expansion, migration, protease, protease inhibitor, metalloproteinase, plasmin, ADAMTS

## Abstract

The migratory and matrix-invading capacities of the cumulus–oocyte complex have been shown to be important for the ovulatory process. In metastatic cancers, these capacities are due to increased expression of proteases, however, there is limited information on protease expression in the cumulus–oocyte complexes. The present study examined cumulus–oocyte complex expression of plasmins, matrix metalloproteases, and A Disintegrin and Metalloproteinase with Thrombospondin Motifs family members in the rat and human. In the rat, human chorionic gonadotropin (hCG) administration increased cumulus–oocyte complex expression of *Mmp2*, *Mmp9*, *Mmp13*, *Mmp14, Mmp16*, *Adamts1*, and the protease inhibitors *Timp1*, *Timp3,* and *Serpine1* by 8–12 h. This ovulatory induction of proteases in vivo could be mimicked by forskolin and ampiregulin treatment of cultured rat cumulus–oocyte complexes with increases observed in *Mmp2*, *Mmp13*, *Mmp14*, *Mmp16*, *Mmp19*, *Plat*, and the protease inhibitors *Timp1*, *Timp3,* and *Serpine1.* Comparison of expression between rat cumulus–oocyte complexes and granulosa cells at the time of ovulation showed decreased *Mmp9* and increased *Mmp13*, *Mmp14*, *Mmp16*, *Adamts1*, *Timp1*, and *Timp3* expression in the cumulus–oocyte complexes. In human, comparison of expression between cumulus and granulosa cells at the time of in vitro fertilization retrieval showed decreased *MMP1*, *MMP2, MMP9*, and *ADAMTS1*, while expression of *MMP16*, *TIMP1,* and *TIMP3* were increased. Treatment of expanding rat cumulus–oocyte complexes with a broad spectrum matrix metalloproteases inhibitor, GM6001, significantly reduced the migration of cumulus cells in vitro. These data provide evidence that multiple proteases and their inhibitors are expressed in the cumulus–oocyte complex and play an important role in imparting the migratory phenotype of the cumulus–oocyte complex at the time of ovulation.

**Summary Sentence**

Multiple proteases and their inhibitors are induced in the cumulus–oocyte complex (COC) during the periovulatory period and potentially play an important role in imparting the migratory phenotype of the COC at the time of ovulation.

## Introduction

The process of ovulation involves multiple events that occur in an intricate, coordinated manner to accomplish the expulsion of the oocyte and surrounding cumulus cells from the preovulatory follicle. The cumulus–oocyte complex (COC) is formed by tight connections through gap-junctional proteins between the oocyte and the specialized granulosa cells surrounding the oocyte called cumulus cells. Unlike the mural and antral granulosa cells that comprise the granulosa cell compartment and line the follicular antrum, cumulus cells do not respond directly to luteinizing hormone (LH) and have decreased LH and progesterone receptor expression [1]. However, under the influence of the mid-cycle gonadotropin surge, the extracellular matrix (ECM) supporting the COC undergoes expansion [1, 2]. This process is brought about by expression of key ECM proteins and proteases within the COC that lead to expansion, detachment, and eventual release of the COC from the surrounding granulosa cells [2]. Disruption of cumulus expansion reduces oocyte release [3–5], demonstrating that COCs actively participate in the ovulatory process [6, 7]. Recent observations that ovulating COCs develop a migratory phenotype similar to that of metastatic cancer cells [7] also support an active role of the COC in oocyte release.

One of the key hallmarks of migratory cells, such as metastatic cancerous cells, is their ability to degrade the surrounding matrix allowing for their migration. This is accomplished by the increased expression of numerous protease systems [8, 9]. A similar situation of increased protease expression is also observed in the ovulatory follicle following the midcycle gonadotropin surge. Proteases derived from the granulosa and theca cell compartments have long been postulated to be critical mediators of the ovulatory process [10–19]. This postulate is supported by the demonstration that administration of specific protease inhibitors or deletion of the A Disintegrin and Metalloproteinase with Thrombospondin Motifs (ADAMTS) 1 gene can block ovulation [19–24].

The predominant proteases induced during the periovulatory period are members of the plasminogen activator (PA)/plasmin, matrix metalloproteinases (MMPs), and ADAMTS families [10–19]. Plasmin is a serine protease that is secreted as a zymogen, known as plasminogen, which must be enzymatically cleaved by tissue-type (tPA or PLAT) or urokinase-type PA (uPA or PLAU) to its active form [11, 25, 26]. Regulation of ovarian proteolysis in the extracellular space occurs through protease inhibitors such as PA inhibitors (PAIs also known as serpines) that are also induced during the periovulatory period [27, 28]. There are two forms of PAI, SERPINE1 (formerly known as PAI1) and SERPINB1 (formerly known as PAI2) [11, 29]. In both the rat and the rhesus macaque, SERPINE1 is predominantly expressed in the theca cells [28, 30, 31] and it acts to inhibit the PA that is secreted by the granulosa cells during the periovulatory period [11]. Inhibition of PA activity by intrabursal injection of α_2_-antiplasmin or antibodies against tPA in rats blocked gonadotropin-induced ovulation [20, 21]. In addition to the PAIs, we have recently observed an hCG induction of the serine protease inhibitor, tissue factor pathway inhibitor 2, in both the human and rodent ovary that was able to inhibit granulosa cell derived plasmin activity [32] suggesting the presence of other plasmin activity modulators.

In addition to the induction of the PA system, LH induces multiple MMPs during the process of ovulation in rats and humans [15, 17, 18, 33]. Proteinases of the MMP family belong to the superfamily of structurally related zinc endopeptidases that are collectively referred to as metzincins and consist of approximately 25 forms of vertebrate MMPs [34]. The importance of the MMPs in the ovulatory process is evident by the induction of these proteinases across species and the ability of MMP inhibitors to block oocyte release in the rat [23, 24] and macaque [19]. Like MMPs, the ADAMTS are a class of 19 related secreted metalloendopeptidases [35]. Among the ADAMTS members, ADAMTS1 is induced by the ovulatory stimulus in mice, rats, and humans [13, 18], and mice lacking ADAMTS1 have a 77% reduction in ovulation [22, 36]. Other studies have reported that in the rat, *Adamts4* mRNA is induced by the ovulatory stimulus whereas ADAMTS5 does not change in response to hCG [37]. In human granulosa cells, there is an increase in the expression of *ADAMTS1* and *9* mRNA following hCG stimulation [18] while in human cumulus cells, *ADAMTS9* was positively associated with embryo viability in PCOS patients [38].

The proteolytic activity of MMPs and ADAMTS is regulated by the TIMPs [39]. There are four distinct members of TIMP family named sequentially as TIMP1, 2, 3, and 4. TIMPs are secreted into the extracellular space and are selective in their ability to inhibit the different MMPs. For example, TIMP2 can regulate the activities of MMP2 and MMP14 while TIMP1 acts to suppress the activity of MMP9 and only weakly inhibits MMP14, 16, and 19 [10, 40]. All TIMPs are expressed in the rat ovary but only the mRNAs for *Timp1*, *2*, and *3* are increased while *Timp4* is decreased during the rat periovulatory period [10, 16]. In the human, *TIMP1* increases after hCG whereas *TIMP2* and *TIMP3* are unchanged ([41], unpublished observations).

Because ovulating COCs manifest a migratory phenotype [7], we hypothesize that these COCs express multiple proteases that instill this migratory phenotype. This is supported by observations that *Adamts1* and *4* mRNAs increase in mouse COCs following ovulation induction [37, 42], *ADAMTS1* and *9* are expressed in human cumulus cells [38, 43], *ADAM17* and *ADAMTS4* are increased in porcine cumulus cells [44–46], *MMP2* increases in bovine cumulus cells [47] and tPA activity increases in the rat ovulating COC [48]. However, there has been no systematic examination of proteases in cumulus cells or comparison of protease expression between the granulosa and cumulus cells between species. Therefore, the importance of this study is the examination of the expression profile of proteases belonging to the plasmin, MMP and ADAMTS family, and their inhibitors in the human and rat COC during the periovulatory period. Furthermore, an in vitro model was used to test the effect of MMP inhibition on cumulus cell migration.

## Methods

All chemicals were sourced from Sigma-Aldrich (St. Louis, MO, USA) or Thermo Fisher Scientific (Pittsburgh, PA, USA) unless otherwise stated.

### Rat COC collection

Immature female Sprague–Dawley rats (Envigo, Indianapolis, IN, USA) were maintained at room temperature with food and water provided ad libitum. Rats were administered 10 IU of pregnant mare serum gonadotropin (PMSG) s.c. at 22–23 days of age to stimulate ovarian follicular development. Forty-eight hours following PMSG injection, five IU of hCG was administered to induce the ovulatory process. For collection of COCs, rats were killed at defined intervals after hCG treatment (0, 4, 8, 12, and 24 h). Ovaries were punctured with a 26G needle to release granulosa cells and COCs into a Petri dish containing DMEM media. The intact COCs were then individually collected under a dissection microscope and transferred into a new dish containing DMEM media. A second round of selection was performed to reduce contamination with granulosa cells. The media containing the COCs was transferred to a microfuge tube, pelleted by centrifugation at 300×g and stored at –70°C until further analysis. Another set of animals were similarly stimulated and sacrificed at 12 h post hCG to collect granulosa cells and COCs as described above. All animal procedures for these experiments were approved by the University of Kentucky Institutional Animal Care and Use Committee.

### In vitro COC expansion

In vitro maturation or expansion of COCs was carried out as described by Eppig et al. [49] with slight modifications. Rats were administered 10 IU of PMSG s.c. at 22–23 days of age, animals were killed and ovaries collected 48 h following PMSG injection. Ovaries from 3 to 4 animals were pooled together to isolate and collect the COCs as described above. This pool was considered as *n* = 1. About 25 intact COCs per well were then placed in a 96 well tissue culture plate in media containing 0.1% bovine serum albumin (BSA), 3 mM glutamate, 25 mM HEPES, and 0.25 mM sodium pyruvate in DMEM. The COCs were then treated with forskolin (FSK, 10 μM) and 250 ng/ml of recombinant mouse amphiregulin (AREG, R & D Systems, Minneapolis, MN, USA) to induce expansion and cultured for 0, 4, 8, 12, or 24 h. Control COCs were also cultured in the absence of FSK and AREG for the same amount of time. At the end of these time points, media was removed and COCs collected to be processed for RNA isolation.

### Human granulosa and cumulus cell collection

Human granulosa cells were collected from patients undergoing in vitro fertilization (IVF) at the Bluegrass Fertility Center, Lexington, KY for non-ovarian etiologies (i.e. male factor infertility, oocyte donors) as described previously [50]. Briefly, women undergoing IVF were administered recombinant human follicle stimulating hormone (FSH) to induce controlled ovarian stimulation. The dose and duration of FSH stimulation was adjusted to achieve maximal follicle growth and maturation as determined by the number of follicles, the follicle size, and estradiol production. After 9–11 days of FSH treatment, patients were given an ovulatory dose of hCG. Granulosa cells and COCs were extracted 36 h post hCG utilizing ultrasound guided needle aspiration. COCs were separated from the aspirate and cumulus cells obtained by exposure to hyaluronidase (80 IU/ml) for up to 1 min and then mechanically dissociated using a 27G needle and 300 μl pipette. The red blood cells from the aspirate were separated by Percoll gradient centrifugation to obtain granulosa cells. Both granulosa and cumulus cells were pelleted separately and stored at –70°C until further analysis. Thus, the human cumulus samples were comprised of only cumulus cells whereas the rat contained the oocyte in a COC. All procedures for these experiments involving human subjects were approved by the University of Kentucky Institutional Review Board. Patient informed consent was obtained from all patients enrolled in this study before utilizing their granulosa cells.

### Real-time RT-PCR

Total RNA was isolated using an RNAeasy kit (Qiagen, Germantown, MD) as per the manufacturer’s guidelines. Total RNA was reverse transcribed into cDNA for use in the gene expression analysis.

The mRNA expression of the different proteases and their inhibitors in the rat COCs were analyzed using either TaqMan or SYBR green-based real-time RT-PCR techniques. The SYBRgreen based real time RT-PCR analysis was used to assess COC gene expression in the rat *in vivo* ovulation induction and *in vitro* COC expansion time course experiments. TaqMan based real time RT-PCR analysis was used to compare the gene expression between granulosa and cumulus cells in both rat and human samples. The following assay-on-demand primer-probe sets from Applied Biosystems (Carlsbad, CA) were used for the TaqMan based gene expression analysis: Rn00579172_m1 (*Mmp14*), Rn00679255_m1 (*Mmp16*), Rn01471830_m1 (*Mmp25*), Rn00820748_g1 (*Rpl32*), Hs00899658_m1 (*MMP1*), Hs01548727_m1 (*MMP2*), Hs00234579_m1 (*MMP9*), Hs01037003_g1 (*MMP14*), Hs00234676_m1 (*MMP16*), Hs00171558_m1 (*TIMP1*), Hs00165949_m1 (*TIMP3*), and Hs01126606_m1 (*SERPINE1*). The target gene and the control gene real time analysis were carried out in the same reaction and the expression levels were normalized to the control gene *Rpl32* in the rat and *GAPDH* in the human.

For SYBRgreen-based assays, oligonucleotide primers corresponding to cDNA for the rat proteases and their inhibitors were designed using PRIMER3 software (Part of Biology Work Bench Version 3.2 available at http://workbench.sdsc.edu/) and the specificity for each primer set was confirmed by PCR product electrophoresis and analyzing the melting (dissociation) curve after each real time PCR reaction. The sequences of the primers used are shown in Table 1. PCR reactions were performed on an Mx3000P 124 QPCR System (Stratagene). The relative amount of each transcript was calculated using the ΔΔC_T_ method and normalized to the endogenous reference gene *Rpl32*.

**Table 1:** Primers Used for Quantitative PCR.

**Gene ID**	**Forward primer**	**Reverse primer**	**Accession number**
Mmp2	AAGAGGCCTGGTTACCCTGT	AAGTAGCACCTGGGAGGGAT	NM_031054.2
Mmp9	TAATAAACACGGATCCCCCA	GGTCAGAACCGACCCTACAA	NM_031055.1
Mmp10	ACCCCACTCACATTCTCC	CCATTTCTCATCATCATCG	NM_133514.1
Mmp13	TGCGGTTCACTTTGAGGACA	TCTTCTATGAGGCGGGGATA	NM_133530.1
Mmp19	AGACCAACCCTCAGCAGCTA	CCAAGACTGATTCCACGGTT	NM_001107159.1
Timp1	CGCTAGAGCAGATACCACG	TTAGCCCTTATAACCAGGTCC	NM_053819.1
Timp3	TCTGCAACTCCGACATCG	GCGTAGTGTTTGGACTGATAGC	NM_012886.2
Adamts1	GCACCTCCGCGGTTCCACAT	CGCGACCCGAGTTGCTGGTT	NM_024400.2
Plat	TCAGTCATGGCTACGTCCCAT	CATCTCGGTTCACTGCAACTTC	NM_013151.2
Serpine1	CAAGAGCAGCTCTCTGTAGCACAA	GCTGAGACTAGAATGGCTGTGGA	NM_012620.1
Rpl32	GAAGCCCAAGATCGTCAAAA	AGGATCTGGCCCTGGCCCTTGAATCT	NM_013226.2

### Western blot analysis

The intact rat COCs were individually collected under a dissection microscope from PMSG:hCG primed rats as described earlier. COCs from 3–4 animals (for 0 h and 12 h time points) were pooled together whereas COCs from 12 animals were pooled for 24-h time point. Whole-cell extracts were isolated by adding sample buffer (x2) and denatured at 95°C for 5 min. Proteins were separated by 12.0% sodium dodecyl sulfate-polyacrylamide gel electrophoresis and subsequently transferred to nitrocellulose membranes. Membranes were blocked for 1 h at room temperature in TBST (25 mM Tris, 137 mM NaCl, 2.7 mM KCl, and 0.05% TWEEN-20) containing 5% low-fat milk and then incubated overnight at 4°C with 1:1000 anti-MMP13 antibody (Abcam Cat# ab39012, RRID:AB_776416), 1:1000 ADAMTS1 antibody (NSJBio Cat#R31182, RRID:AB_3075893), and 1:1000 rabbit anti-BACT (13E5; Cell Signaling Technology Cat# 4970, RRID:AB_2223172). The membranes were incubated with 1:2000 secondary horseradish peroxidase–conjugated antibody (Cell Signaling Technology Cat#7074, RRID: AB_2099233) for 1 h. A chemiluminescent signal was generated with the Amersham ECL Prime Western blotting detection reagent and the signal was captured using ChemiDoc (Bio-Rad Laboratories, CA).

### Cumulus cell migration

The effect of MMP inhibition on migration of rat cumulus cells was assessed by determining the number of cumulus cells that migrated through a 12 μm pore sized transmembrane insert and ended up at the bottom of the cell culture plate. Briefly, 24-well flat bottom Costar cell culture plates (Corning Life Sciences, Kennebunk, ME) were rehydrated with warm serum free COC collection media (0.1% bovine serum albumin (BSA), 3 mM glutamate, 25 mM HEPES, 0.25 mM sodium pyruvate, and 1% gentamycin in αMEM). Round glass coverslips were placed at the bottom of the culture plate. Intact rat COCs were obtained from ovaries at 4 h of post hCG stimulation as described above. COCs were pooled from 4 ovaries and 30–35 intact COCs were plated (in 300 μl) in the upper chamber of the insert of the cell culture plate. This pool is considered as n = 1. The bottom well contained COC collection media with or without epidermal like-growth factor (EGF, 10 ng/ml, serving as positive chemoattractant) in the presence or absence of GM6001, a broad-spectrum MMP inhibitor (25 μM). As GM6001 has a Ki of 0.4 nM against fibroblast collagenase, 0.1 nM against neutrophil collagenase, 0.2–0.5 nM against gelatinases, and 27 nM against stromelysin [51] it was expected that the dose of 25 μM used could lead to effective neutralization of multiple MMP and ADAMTS proteases. As a control, the bottom well contained COC collection media with addition of 10% FBS. Of note, this approach assesses migration through transwell pores rather than invasion through a barrier ECM.

After 24 h culture, the inserts were removed and cells adhered on to the glass coverslip on the bottom of each well were fixed in 4% paraformaldehyde. Then, cells adhered on the glass coverslip were stained with DAPI (Sigma-Aldrich), the glass coverslip was mounted upside down on microscope slides, and images were captured using an Eclipse E800 Nikon microscope. The cumulus cells were counted from the 6–8 image sections of the coverslip (N = 4) to estimate the number of cells that migrated through the transmembrane insert.

### Statistical analysis

All data were checked for normalcy of variance using Bartlett’s chi-square test. Data not normally distributed were log transformed for analysis. Changes in gene expression during the rat *in vivo* time course experiment were analyzed by one-way analysis of variance (ANOVA) and followed by Bonferroni post-hoc tests. The changes in gene expression in the in vitro COC expansion experiment were analyzed by two-way ANOVA, followed by Bonferroni’s multiple comparison tests. The gene expression data from rat and human granulosa and cumulus cells were log transformed and analyzed using a Student’s t-test. The migration data were not log transformed and were analyzed by ANOVA and a Tukey’s posthoc test. All analyses were performed using Prism (GraphPad Software Inc, La Jolla, CA; Version 5.00). Differences were considered significant if the p-value was <0.05.

## Results

### Proteases and their inhibitors induced in the rat COC during the periovulatory period in vivo

**Figure 1:**
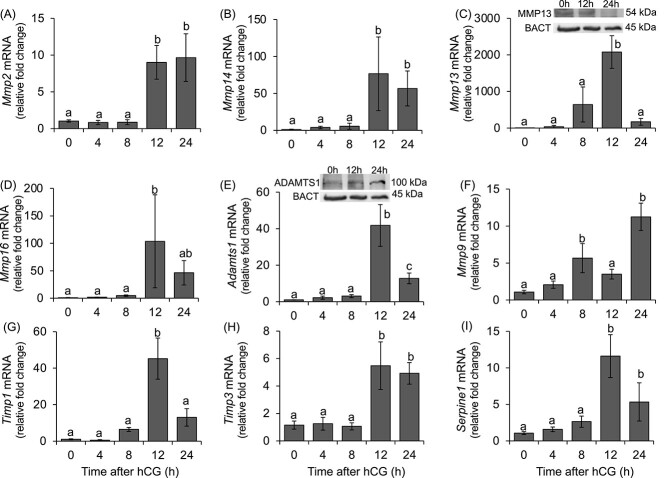
Expression profile of proteases and protease inhibitors in the rat COC during the periovulatory period in vivo. Depicted is the mRNA expression profile of the proteases *Mmp2* (A), *Mmp14* (B), *Mmp13* (C), *Mmp16* (D), *Adamts1* (E), and *Mmp9* (F) and the protease inhibitors *Timp1* (G), *Timp3* (H), and *Serpine1* (I) in the COC collected from PMSG-primed immature rats at 0 (48 h after PMSG), or 4, 8, 12, and 24 h after administration of hCG. Relative levels of mRNA were normalized to *Rpl32* in each sample and expressed as a fold change relative to the 0 h (*n* = 4/time point). Different superscripts indicate significant changes (*P* < 0.05) in mRNA levels. Representative western blots of MMP13 (inset, upper panel C), ADAMTS1 (inset, upper panel E), and BACT proteins as loading control at 0, 12, and 24 h.

The mRNA expression of proteases and protease inhibitors that are known to be induced in the ovary [15–19] were examined in rat COCs after administration of an ovulatory dose of hCG *in vivo*. These time points correlate with complete COC expansion from no expansion (0, 4 h hCG), partial expansion (8, 12 h hCG) to full expansion (24 h hCG, [52]). The COC protease mRNA expression exhibited 3 distinct patterns. In the first pattern, there was no change in MMP expression during the early ovulatory period from 0 to 8 h after hCG administration but expression increased dramatically by 12 h and remained elevated in the postovulatory period through 24 h. This pattern was observed for *Mmp2* and *Mmp14* (Figure 1A and 1B respectively). In the second pattern, the MMP mRNA expression was low from 0 to 8 h post hCG, increased by 12 h, but declined to pre-hCG levels by 24 h. This pattern was observed for *Mmp13* and *Mmp16* (Figure 1C and 1D respectively). Likewise, Mmp13 protein as shown by Western blot also showed similar pattern of expression where Mmp13 was highest at 12 h and decreased by 24 h (Figure 1C. inset). The expression of *Adamts1* was similar to the second pattern but at 24 h declined to a level intermediate between 12 h and pre-hCG levels (Figure 1E). Also, Adamts1 protein levels were elevated at 12 h but unlike mRNA expression the protein level remained sustained until 24 h (Figure 1E inset). The third pattern was a bimodal induction where mRNA expression was increased by 8 h post hCG, declined at 12 h and increased again at 24 h post hCG. This pattern was observed for *Mmp9* (Figure 1F). The mRNA expression of proteases *Mmp10*, *Mmp19*, *Mmp25* and *Plat* were also examined but did not show any change after hCG administration in the rat COCs *in vivo* (data not shown).

The mRNA expression of protease inhibitors, *Timp1*, *Timp3*, *Serpine1,* and *Tfpi2* were also examined in rat COCs after administration of an ovulatory dose of hCG in vivo. The expression pattern for the protease inhibitors followed two of the patterns observed for the proteases. The mRNA expression of *Timp1* was low from 0 to 8 h and dramatically increased by 12 h before declining by 24 h post-hCG administration (Figure 1G). In contrast, the mRNA expression of both *Timp3* and *Serpine1* were low from 0 to 8 h, increased by 12 h and remained elevated through 24 h post-hCG administration (Figure 1H and I). The mRNA expression of the serine protease inhibitor, *Tfpi2,* did not show any change before nor after hCG administration (data not shown).

### Expression of proteases and their inhibitors in cumulus cells and granulosa cells during the periovulatory period in vivo

**Figure 2:**
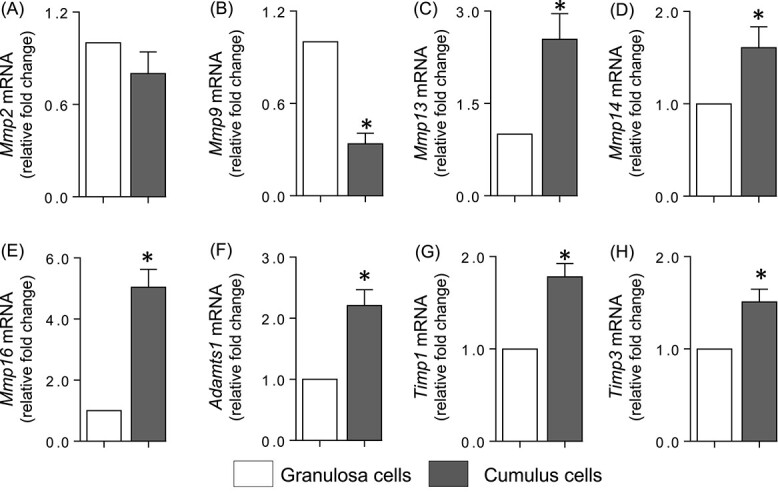
Expression profile of proteases and their inhibitors in rat granulosa cells and COCs in vivo. Depicted is the mRNA expression profile of the proteases *Mmp2* (A), *Mmp9* (B), *Mmp13* (C), *Mmp14* (D), *Mmp16* (E), *Adamts1* (F), and protease inhibitors *Timp1* (G) and *Timp3* (H) in granulosa and cumulus cells collected from the same animal at 12 h post hCG administration. Relative levels of mRNA were normalized to *Rpl32* in each sample and expressed as a fold change relative to granulosa cells (*n* = 5). * indicates significant statistical difference (*P* < 0.05) in mRNA levels as determined by Student’s *t*-test.

The mRNA levels of granulosa and cumulus cells were compared just before ovulation to assess the relative abundance of the proteases and their inhibitors in the COC compared to the granulosa compartment in both the rat and human. In the rat, expression was examined in the granulosa cells and COCs collected 12 h after administration of an ovulatory dose of hCG to PMSG-primed immature rats, a time point immediately prior to ovulation. While there was no difference in the *Mmp2* mRNA expression (Figure 2A), *Mmp9* mRNA levels were lower in cumulus cells compared to granulosa cells (Figure 2B). In contrast, the expression of *Mmp13*, *14* and *16* and *Adamts1* were higher in the cumulus cells (Figure 2C–F). Similarly, expression of *Timp1* and *3* (Figure 2G and H) were also higher in cumulus cells, while there was no change in *Serpine1* mRNA (data not shown).

**Figure 3:**
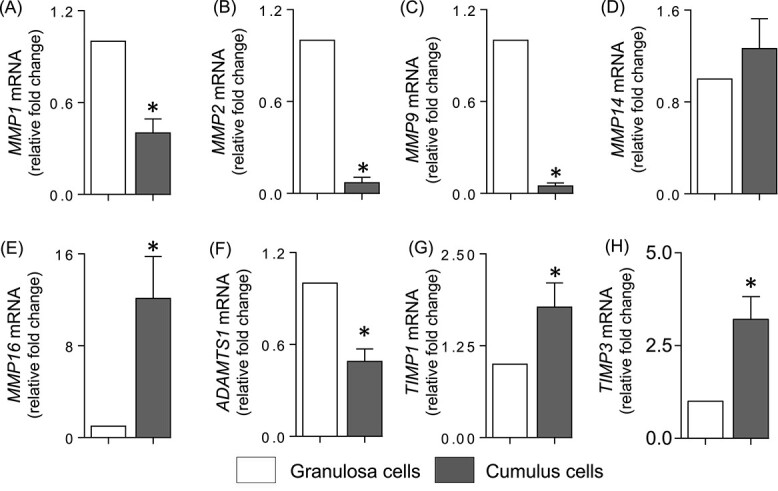
Expression profile of the proteases and their inhibitors in human granulosa and cumulus cells in vivo. Depicted is the expression profile of the proteases *MMP1* (A), *MMP2* (B), *MMP9* (C), *MMP14* (D), *MMP16* (E), and *ADAMTS1* (F), and protease inhibitors *TIMP1* (G) and *TIMP3* (H) in granulosa and cumulus cells collected 36 h post hCG administration from women undergoing IVF. Relative levels of mRNA were normalized to *GAPDH* in each sample and expressed as a fold change relative to the expression in granulosa cells (*n* = 4–8). Granulosa and cumulus cell expression were compared from the same patient. * indicates significant statistical difference (*P* < 0.05) in mRNA levels as determined by Student’s *t*-test.

To compare the protease and inhibitor mRNA expression profiles in different ovarian compartments between rat and human, human cumulus cells and granulosa cells were collected 36 h post hCG in patients undergoing IVF. Expression of *MMP1*, *2*, *9* (Figure 3A–C) and *ADAMTS1* (Figure 3F) was lower in cumulus cells compared to granulosa cells. In contrast, *MMP14* expression did not change (Figure 3D) while *MMP16* expression was significantly higher (Figure 3E) in cumulus cells. Among the protease inhibitors, *TIMP1* and *TIMP3* mRNA were higher in the cumulus compared to granulosa cells (Figure 3G and H), while there was no change in *SERPINE1* mRNA (data not shown).

### Proteases and protease inhibitors induced in rat COCs during in vitro expansion

**Figure 4:**
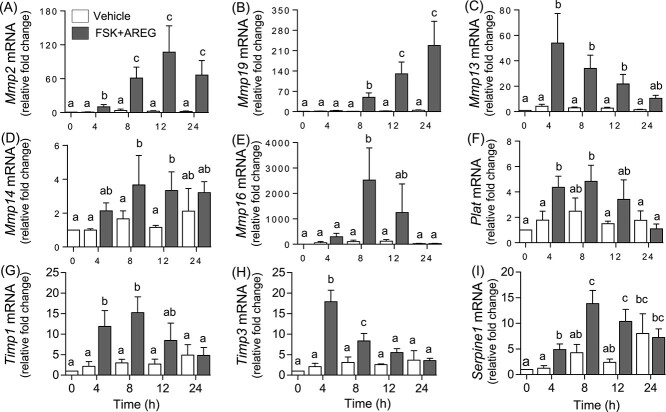
Expression profile of proteases and protease inhibitors following in vitro induced COC expansion. COCs collected from PMSG-primed immature rats were cultured in the presence (solid bars) or absence (open bars) of FSK + AREG for 0, 4, 8, 12, and 24 h and processed for mRNA analysis. The mRNA expression profile of the proteases *Mmp2* (A), *Mmp19* (B), *Mmp13* (C), *Mmp14* (D), *Mmp16* (E), and *Plat* (F) and the protease inhibitors *Timp1* (G), *Timp3* (H), and *Serpine1* (I) are shown. Relative levels of mRNA were normalized to *Rpl32* in each sample and expressed as a fold change relative to the 0 h (*n* = 4/time point). Different superscripts indicate significant changes (*P* < 0.05) in mRNA levels.

The mRNA expression of proteases was examined in rat COCs induced to undergo expansion *in vitro*. Unlike the expression profile for proteases *in vivo*, two distinct patterns were observed. In the first pattern, FSK + AREG induced protease mRNA expression within 4-8 h, which then remained elevated or increased by 24 h after treatment. This pattern was observed for *Mmp2* and *Mmp19* mRNA (Figure 4A and 4B respectively). In the second pattern, FSK + AREG induced protease mRNA expression within 4-8 h, which then declined by 24 h to basal levels. This pattern was observed for *Mmp13, Mmp14, Mmp16,* and *Plat* mRNA (Figure 4C–F, respectively). The mRNA expression of the proteases did not change in vehicle treated COCs across the 24-h time period examined. The mRNA expression of *Mmp9*, *Mmp25,* and *Adamts1* was not induced by FSK + AREG (data not shown).

The mRNA expression of the protease inhibitors *Timp1*, *Timp3*, *Serpine1,* and *Tfpi2* was also examined during *in vitro* induced expansion of rat COCs. Again, two distinct patterns were observed. The expression of *Timp1* and *Timp3* was induced by FSK + AREG within 4 h which then declined by 24 h to basal levels (Figure 4G and 4H respectively). In the second pattern, FSK + AREG induced protease inhibitor mRNA expression within 4 h which then remained elevated at 24 h after treatment. This pattern was also observed for *Serpine1* mRNA (Figure 4I). However, in contrast to the other protease inhibitors, the *Serpine1* mRNA levels in the rat COCs treated with vehicle did not change from 0 to 12 h but showed an increase by 24 h of culture (Figure 4I). The mRNA expression of serine protease inhibitor *Tfpi2* did not show any changes with FSK + AREG treatment (data not shown).

### Effect of broad-spectrum MMP inhibitor treatment on cumulus cell migration

**Figure 5:**
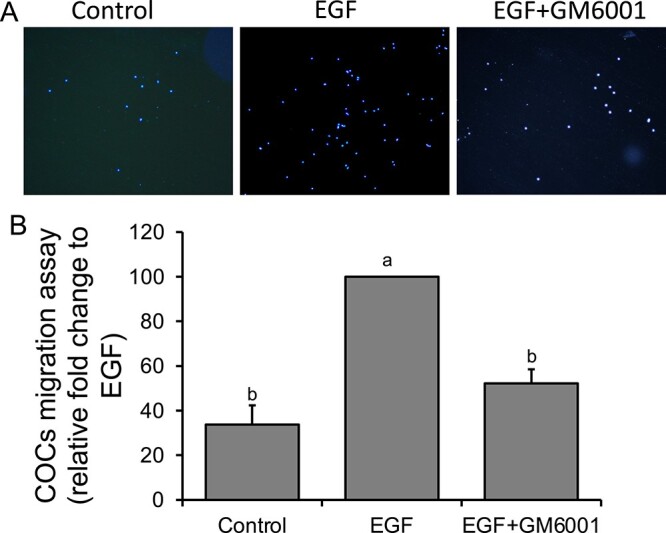
Effect of a broad-spectrum MMP inhibitor on rat cumulus cell migration. (A) Representative images of rat cumulus cell migration. Cells were treated with or without EGF as a positive chemoattractant in the presence or absence of the broad-spectrum MMP inhibitor, GM6001. Cumulus cells that migrated through a 12 μm transmembrane and attached to round glass coverslips at the bottom of the well were fixed and stained with DAPI. (B) Bar graph showing the percentage of cumulus cells that migrated through the transmembrane to the glass coverslip. Cell migration was normalized to the EGF treatment and set to 100% (*n* = 4). Different superscripts indicate significant changes (*P* < 0.05).

Rat COCs were cultured with media containing FBS (control) or EGF (positive control) with or without the broad-spectrum MMP inhibitor, GM6001 for 24 h, to assess if inhibition of MMP activity altered cumulus cell migration. The representative images of cumulus cells that migrated through the membrane and adhered to the glass coverslip at the bottom of the well shows the difference in the cell number among the treatments (Figure 5A). As shown in Figure 5B, EGF treatment increased cumulus cell migration by 3-fold compared to the control cells. Addition of GM6001 in the presence of EGF significantly reduced cumulus cell migration by 48% compared to those that did not receive GM6001 treatment (Figure 5B).

## Discussion

The ovarian extracellular matrix undergoes dynamic changes during the periovulatory period and these changes are tightly regulated by the expression of proteases and their inhibitors [10, 11, 13]. The expression of these proteases and their inhibitors is found in numerous different ovarian cells such as the granulosa, theca, and stromal cells of many species including the rat and human [10–19, 53, 54]. However, limited studies have examined the expression of proteases in the cumulus cells. These previous studies report that in cumulus cells *MMP2* is present in the bovine, *ADAMTS1* is expressed in human, porcine, and mouse, *ADAMTS9* is found in the human, and *ADAMTS4* and *5* are present in the mouse [37, 38, 42–44, 47].

Here we demonstrate for the first time that in rat cumulus cells, multiple members of the MMP family such as *Mmp2*, *9*, *13*, *14*, and *16* mRNA are increased during the periovulatory period along with the expression of the protease inhibitors *Timp1*, *Timp3,* and *Serprine1*. We also compared the expression between granulosa and cumulus cells of these proteases and their inhibitors in both the rat and human. In the granulosa cells, we found that the collagenase *MMP1* was highly expressed in the human whereas the gelatinases were abundant in the human (*MMP2* and *9*) and rat (*Mmp9*). In the cumulus cells, the membrane type-matrix metalloproteinases (MT-MMPs) were highly expressed in the human (*MMP14* and *16*) and rat (*Mmp16*) along with the MMP inhibitors (*TIMP1* and *3* in both species).

The current findings are in agreement with the work of Luddi and coworkers [53], who determined the expression of MMP and TIMP members in cumulus and granulosa cells in fertile and infertile women. Similar to our findings, they observed that expression of *MMP2* and *MMP9* was elevated in granulosa cells compared to cumulus cells. Of interest was the observation that *MMP2* expression was elevated in the granulosa cells of older women as well as in cumulus cells from infertile poor responders [53]. It was proposed that this elevated expression may be related to ovarian aging [53]. In contrast to the gelatinases, expression of the stromelysin *MMP11* as well as *TIMP2* was approximately 3-fold higher in cumulus cells than granulosa cells whereas *TIMP1* was unchanged. However, we observe that both *TIMP1* and *TIMP3* were elevated in cumulus cells. Our studies expand upon these previous findings and explore the changes in the membrane type MMPs as well as the ADAMTS.

The expression patterns of specific MMPs are consistent with their potential role and the distribution of their target proteins in the ovarian follicle. In the human, rat, and mouse, the follicle is composed of collagens, fibronectin, and laminin [41, 55–57]. These ECM proteins are the main proteolytic targets of collagenases and gelatinases [10], where the granulosa cell expression of the proteases aid in breakdown of the follicular wall ECM to facilitate the ovulatory process. In contrast, the MT-MMPs are highly expressed in the cumulus cells/COCs. Due to their presence on the cell membrane, the MT-MMPs may act to direct pericellular proteolysis that results in cell expansion and migration [58]. The increased expression of MT-MMPs and TIMP1 could also bring about increased activation of MMP2, as described in rat granulosa cells [59] and other cell types [60]. As the migratory COCs reach the follicular wall, the MMP14–TIMP1 complex may therefore activate the granulosa or theca secreted MMP2 to cause rapid breakdown of the follicular apex and extrusion of the oocyte. Conversely, the increased expression of TIMPs and SERPINE1 in the COCs may protect the complex from granulosa, thecal, or oviductal proteases during the ovulatory process and post ovulation. For example, elevated levels of MMP9 appear to detrimental to bovine oocyte development and maturation [61].

During the process of ovulation, the COC undergoes expansion or mucification that involves synthesis of an ECM. The COC ECM backbone is comprised of glycosaminoglycan and hyaluranon, together with crosslinking proteins such as heavy chains of inter-*a* trypsin inhibitor (IaI) family, tumor necrosis factor-induced protein-6 (Tnfip-6), pentraxin 3 (PTX3), and the proteoglycan versican [6]. Hyaluranon is known to stimulate expression of various MMPs including MMP2 and MMP9 in skin fibroblasts, keratinocytes, and lung cancer cells [62, 63]. Because hyaluranon synthase is increased in the cumulus cells following ovulation induction and cumulus cells express receptors for hyaluranon, CD44 [64], the increased expression of MMPs observed in the cumulus cells may result from the hyaluranon–CD44 interaction. Additionally, the hyaluranon–CD44 binding has also been reported to transactivate EGF receptor [65] and EGF-like ligands are known to promote COC expansion and cumulus gene expression [66]. Therefore, it is possible that the ovulatory stimulus induced EGF-like ligands can either directly or indirectly regulate the expression of proteases in the cumulus cells. Alternatively, this EGF regulation of protease expression may result in a feedback loop where proteases stimulated by EGF in turn regulate EGF actions. For example, ADAM17 is induced in porcine COC and is involved in COC expansion as well as oocyte meiotic maturation through the activation of EGF receptor in cumulus cells [45, 46]. Additional studies are needed to clearly identify these regulators and their interactions.

Consistent with our hypothesis that the migratory phenotype in the COC is associated with proteases, we found a significant reduction in cumulus cell migration in the presence of the broad-spectrum MMP inhibitor GM6001. This inhibitor not only inhibits MMP family members but also members of the ADAMTS family [67]. Cell migration is crucial for the normal physiology and pathology in various organ systems and is influenced by various factors especially the ECM [68]. As proteases regulate ECM turnover, it is no surprise that inhibition of their activity leads to a reduction in cumulus cell migratory capacity.

While blocking protease activity can lead to reduction in cumulus migration, the associated mechanism through which these proteases regulate COC migration is not clear. The COC ECM produced as a result of expansion contains hyaluronan and versican. The presence of hyaluronan and versican in the pericellular matrix has been reported to promote prostate cancer cell mobility [69–71]. Because the migratory phenotype of COCs is associated with changes in its adhesion property to different ECM matrix contained in the follicular wall [7], it is suggested that the ADAMTS1-dependent cleavage of versican may be required to modulate this event [13]. Likewise, it is expected that other proteases identified in the ovulating COCs may influence the adhesion and aid in the migratory capacity by altering the ECM or may promote the proteolytic cleavage of the follicular wall to undergo expulsion as part of the ovulatory process.

In conclusion, the present results provide evidence that ovulating COCs express multiple proteases belonging to the MMP, ADAMTS, and plasmin families and blocking MMP/ADAMTS activity reduces cumulus cell migration. These data provide support for the role of MMP/ADAMTS proteases in inducing the migratory phenotype in the COC and may form one of the many processes that regulate and participate in COC expulsion to bring about successful ovulation.

## Data Availability

Data are available on request.
